# Immune Responses and Viral Persistence in Simian/Human Immunodeficiency Virus SHIV.C.CH848-Infected Rhesus Macaques

**DOI:** 10.1128/JVI.02198-20

**Published:** 2021-04-12

**Authors:** Widade Ziani, Anya Bauer, Hong Lu, Xiaolei Wang, Xueling Wu, Katharine J. Bar, Hui Li, Dongfang Liu, George M. Shaw, Ronald S. Veazey, Huanbin Xu

**Affiliations:** a Tulane National Primate Research Center, Tulane University School of Medicine, Covington, Louisiana, USA; b Department of Medicine, University of Pennsylvania, Philadelphia, Pennsylvania, USA; c Aaron Diamond AIDS Research Center, Columbia University Vagelos College of Physicians and Surgeons, New York, New York, USA; d Department of Pathology, Immunology and Laboratory Medicine, Rutgers University-New Jersey Medical School, Newark, New Jersey, USA; Ulm University Medical Center

**Keywords:** SHIV, persistent infection, viral reservoirs, autologous neutralizing Abs and evolution, autologous neutralizing Abs, evolution

## Abstract

Chimeric simian/human immunodeficiency viruses (SHIVs) are widely used in nonhuman primate models to recapitulate human immunodeficiency virus (HIV) infection in humans, yet most SHIVs fail to establish persistent viral infection. We investigated immunological and virological events in rhesus macaques infected with the newly developed SHIV.C.CH848 (SHIVC) and treated with combined antiretroviral therapy (cART). Similar to HIV/simian immunodeficiency virus (SIV) infection, SHIV.C.CH848 infection established viral reservoirs in CD4^+^ T cells and myeloid cells, accompanied by productive infection and depletion of CD4^+^ T cells in systemic and lymphoid tissues throughout SHIV infection. Despite 6 months of cART-suppressed viral replication, integrated proviral DNA levels remained stable, especially in CD4^+^ T cells, and the viral rebound was also observed after ART interruption. Autologous neutralizing antibodies to the parental HIV-1 strain CH848 were detected, with limited viral evolution at 5 months postinfection. In comparison, heterogenous neutralizing antibodies in SHIV.C.CH848-infected macaques were not detected except for 1 (1 of 10) animal at 2 years postinfection. These findings suggest that SHIV.C.CH848, a novel class of transmitted/founder SHIVs, can establish sustained viremia and viral reservoirs in rhesus macaques with clinical immunodeficiency consequences, providing a valuable SHIV model for HIV research.

**IMPORTANCE** SHIVs have been extensively used in a nonhuman primate (NHP) model for HIV research. In this study, we investigated viral reservoirs in tissues and immune responses in an NHP model inoculated with newly generated transmitted/founder HIV-1 clade C-based SHIV.C.CH848. The data show that transmitted founder (T/F) SHIVC infection of macaques more closely recapitulates the virological and clinical features of HIV infection, including persistent viremia and viral rebound once antiretroviral therapy is discontinued. These results suggest this CCR5-tropic, SHIVC strain is valuable for testing responses to HIV vaccines and therapeutics.

## Abstract (precis)

SHIVs have been extensively used in a nonhuman primate (NHP) model for HIV research. In this study, we investigated viral reservoirs in tissues and immune responses in an NHP model inoculated with newly generated transmitted/founder HIV-1 clade C-based SHIV.C.CH848.

## INTRODUCTION

Chimeric simian/human immunodeficiency viruses (SHIVs), carrying human immunodeficiency virus (HIV) envelope glycoproteins from transmitted founder (T/F) viruses, are invaluable for testing HIV transmission, pathogenesis, and prevention strategies in nonhuman primate (NHP) models (1–5). However, the majority of current SHIVs have limitations, including differences in coreceptor usage and a lack of sustained viremia or progression to AIDS (5–11). Therefore, the development of functional SHIV clones that better mimic the natural history of HIV infection and that reflect coreceptor usage of globally circulating T/F viruses resulting in establishment of persistent viral reservoirs in animal models is of great significance for testing HIV prevention and cure strategies.

Clade C viruses represent the predominant HIV subtype in the global HIV pandemic, yet most SHIVs to date either have been derived from clade B (SHIVsf262P and SHIVAD8) or utilize different coreceptors from that of HIV T/F strains (SHIV89.6P, SHIV KU, etc.), which exclusively utilize CCR5. The new CCR5-tropic clade C SHIV (SHIV.C.CH848 [SHIVC]) clone (12–14) encodes a clade C Env isolated from an acutely infected Malawian man in 2008 (15) and was developed by a single amino acid substitution at Env residue 375 to increase the affinity of CH848 Env for rhesus CD4 (16, 17). In this study, we investigated immunological and virological events in SHIV.C.CH848-infected animals in acute infection and on antiretroviral therapy, including CD4^+^ T cells, neutralizing antibody (Ab) responses, and Env viral evolution. SHIV.C.CH848 infection resulted in acute depletion of peripheral CD4^+^ T cells and persistent viral infection, as indicated by detectable proviral DNA even after 6 months of combined antiretroviral therapy (cART) treatment, and viral rebound after cART interruption. A potent autologous (but not heterogenous) neutralizing antibody response was detected from 5 months to 2 years postinfection (p.i.). These findings suggest that this novel T/F SHIV.C.CH848 is a promising model for HIV latency and cure studies.

## RESULTS

### Plasma viral load and immunological responses in SHIV.C.CH848-infected rhesus macaques on antiretroviral therapy.

**FIG 1:**
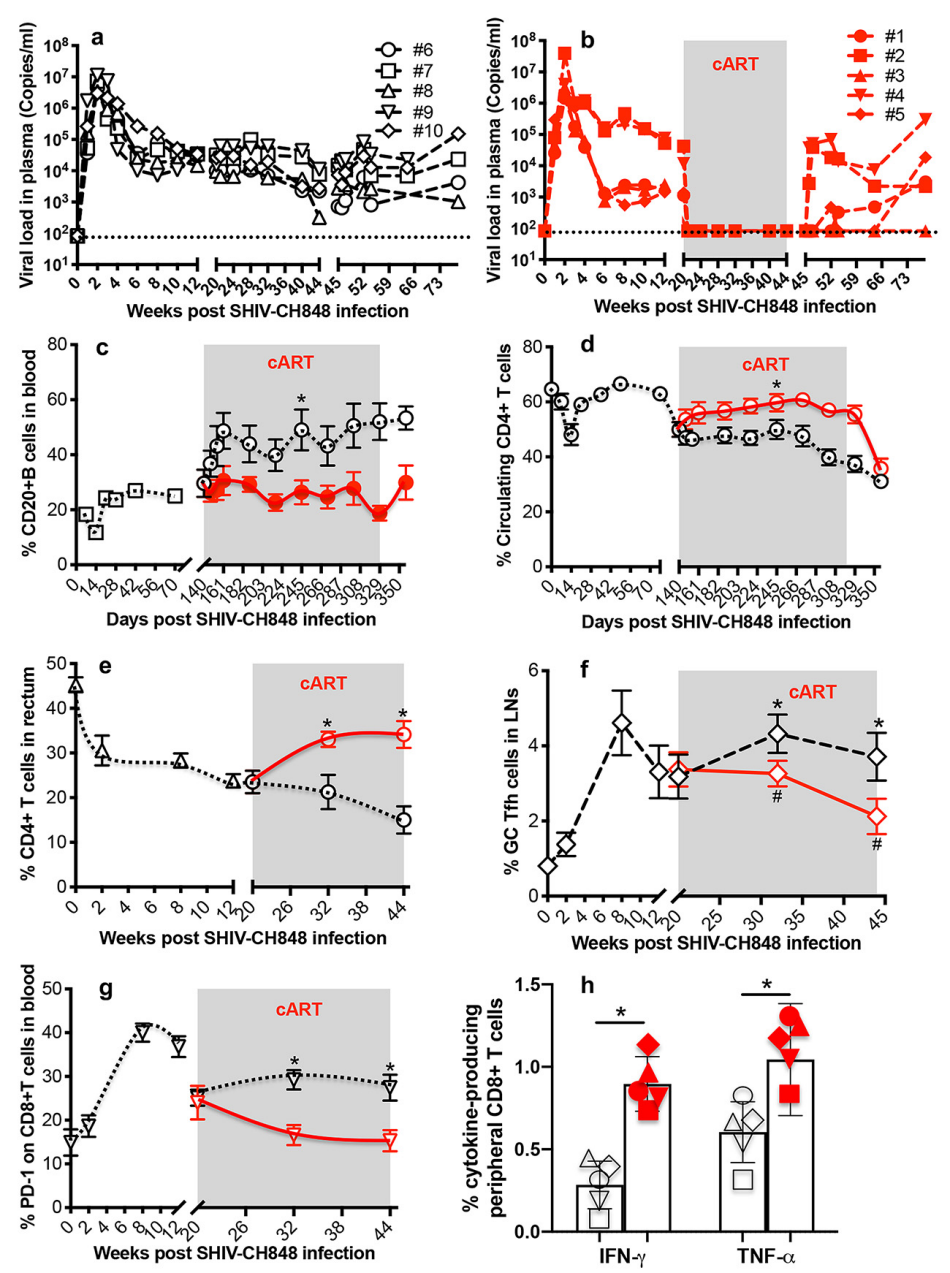
Plasma viral load and immunological events in SHIV.C.CH848-inoculated rhesus macaques on antiretroviral therapy. (a and b) Plasma viral load in SHIV-infected macaques subsequently treated with anti-HIV drugs for 6 months, initiated 5 months post-SHIV infection (*n* = 5 [a]), compared to untreated controls (*n* = 5 [b]). (c to f) Changes in peripheral CD20^+^ B and CD4^+^ T cells, rectal CD4^+^ T cells, and T follicular helper cells in untreated and treated animal groups. (g) PD-1 expression on peripheral CD8^+^ T cells. Note that viral rebound was observed in 4 of 5 animals after cART interruption. (h) SHIV.C.CH848 *gag*-specific cytokine (TNF‐α and IFN‐γ) responses of peripheral CD8^+^ T cells in two animal cohorts with (red) or without ART (black) at 44 weeks post-SHIV infection. Error bars indicate SEM. Paired *t* tests were used to compare ART-treated with untreated groups. *, *P* < 0.01.

Ten rhesus macaques (RMs) were intravenously inoculated with SHIV.C.CH848, and half of the animals received anti-HIV drugs 5 months postinfection. As indicated in Fig. 1a and b, plasma viral load peaked 14 days post-SHIV infection, followed by a relatively sustained viral set point. Once cART was initiated (5 months postinfection), viremia in treated animals (*n* = 5) rapidly declined to undetectable levels after 2 weeks, compared with untreated controls (*n* = 5) that in large part maintained the set point viral load. Notably, viral rebound was detected in 4/5 of the animals after cART interruption, except for 1 animal that remained aviremic up to 6 months after cART cessation. Further, SHIVC infection resulted in significant depletion of peripheral CD4^+^ T cells by 14 days postinfection and progressive reductions of rectal CD4^+^ T cells while peripheral CD20^+^ B cells and GC T follicular help (Tfh) cells (PD-1^high^ CXCR5^+^ cells gated CD4^+^ T cells) expanded in untreated animals by 5 months postinfection. In contrast, cART-treated animals significantly recovered CD4^+^ T cells in blood and rectum while maintaining baseline levels of peripheral CD20^+^ B cells and GC Tfh cells (Fig. 1c to f). Since PD-1 upregulation reflects CD8^+^ T cell exhaustion during viral infection (18, 19), we examined PD-1 expression on peripheral CD8^+^ T cells throughout SHIVC infection. The results showed that SHIVC infection significantly upregulated PD-1 on CD8^+^ T cells in untreated macaques, whereas reduced frequencies of PD-1^+^ CD8^+^ T cells were detected in cART-treated animals (Fig. 1g), consistent with lower simian immunodeficiency virus (SIV) Gag-specific cytotoxic T lymphocyte (CTL) responses in untreated animals at 44 weeks postinfection (Fig. 1h). These data demonstrate that SHIV.C.CH848 recapitulates key virological and immunological characteristics of HIV-1 infection.

### Viral dissemination in systemic and lymphoid compartments of SHIV.C.CH848-infected rhesus macaques on antiretroviral therapy.

**FIG 2:**
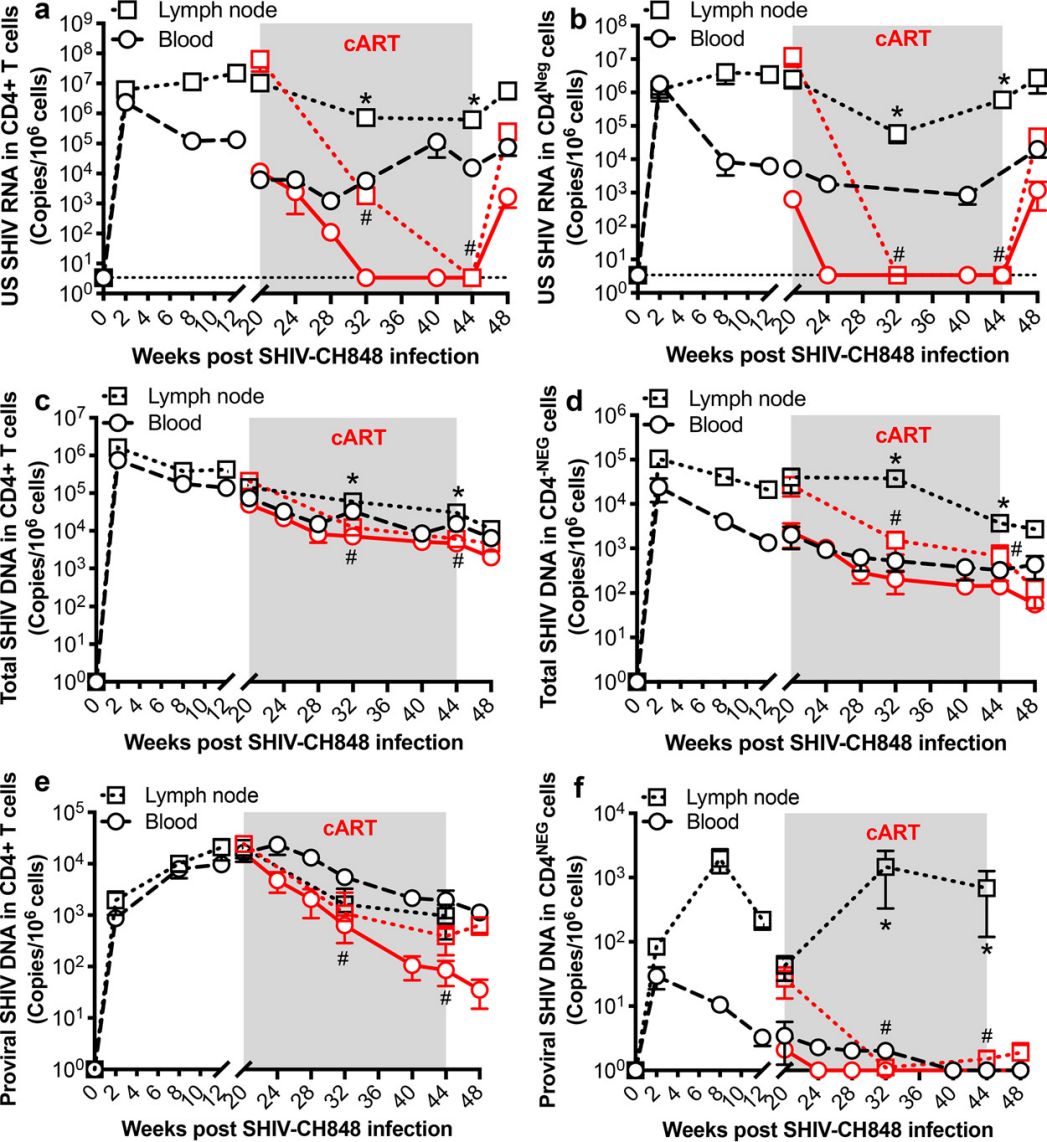
Dynamics of cell-associated SHIV RNA and DNA in CD4^+^ T cells from PBMCs and lymph nodes of SHIV.C.CH848-infected macaques on antiretroviral therapy, compared with CD4-negative cells. Shown are results of longitudinal analysis of unspliced SHIV RNA (US SHIV RNA) (a and b), total SHIV DNA (c and d), or integrated proviral SHIV DNA (e and f) in CD4^+^ T cells and CD4-negative cell populations derived from peripheral blood and lymph nodes of SHIV-infected macaques with or without cART. Note that proviral DNA was detectable even under cART, especially in purified CD4^+^ T cells. Cell-associated SHIV RNA and DNA are expressed as copies per 1 million cells. The dotted line represents the limit of detection (LOD) calculated as described in Materials and Methods. Error bars indicate SEM. *, *P < *0.01, determined by two-tailed paired *t* test in either blood or lymph node tissue of treated and untreated animals; #, *P* < 0.01, compared between pretreatment (month 5 p.i.) and after treatment in both tissues at different time points.

To evaluate the dynamics of cell-associated SHIVC RNA/DNA in systemic and lymphoid tissues after SHIVC infection and their response to cART, unspliced (US) SHIVC RNA, total SHIV DNA, and proviral DNA were longitudinally measured in blood, lymph node (LN), and rectal biopsy specimens from SHIVC-infected macaques. As CD4^+^ T cells are preferentially targeted in HIV infection, CD4^+^ T cells were also purified from blood and lymph nodes to further assess cell-associated SHIV RNA/DNA levels, compared with the remaining CD4-negative cell populations. As shown in Fig. 2, both SHIV RNA and DNA were detected in both CD4^+^ and CD4-negative cell populations in peripheral blood mononuclear cells (PBMCs) and lymph node-derived mononuclear cells. Antiretroviral therapy suppressed SHIVC replication in both cell populations in blood and lymph nodes compared with that in untreated controls. Levels of US SHIVC RNA decreased to undetectable levels in both cell populations by 3 months of cART treatment, although LN-derived CD4^+^ T cells still had detectable viral RNA (vRNA) at this time point. At 1 month of cART interruption, US SHIVC RNA increased in both cell populations (Fig. 2a and b). Similarly, cART also reduced levels of total SHIVC DNA and proviral DNA in both CD4^+^ and CD4-negative cell populations within 3 months of treatment (Fig. 2c to f). However, total and proviral DNAs were still detectable in CD4^+^ T cells throughout SHIVC infection, regardless of treatment duration. Notably, there was no significant reduction of proviral DNA levels in LN-derived CD4^+^ T cells in treated animals. However, there was no detectable proviral DNA in the corresponding CD4-negative cells after treatment (Fig. 2e and f). These data show that CD4^+^ T cells are the major reservoir for SHIV.C.CH848 persistence, especially in organized lymphoid tissues (LN), mimicking the hallmark qualities of HIV infection in humans.

**FIG 3:**
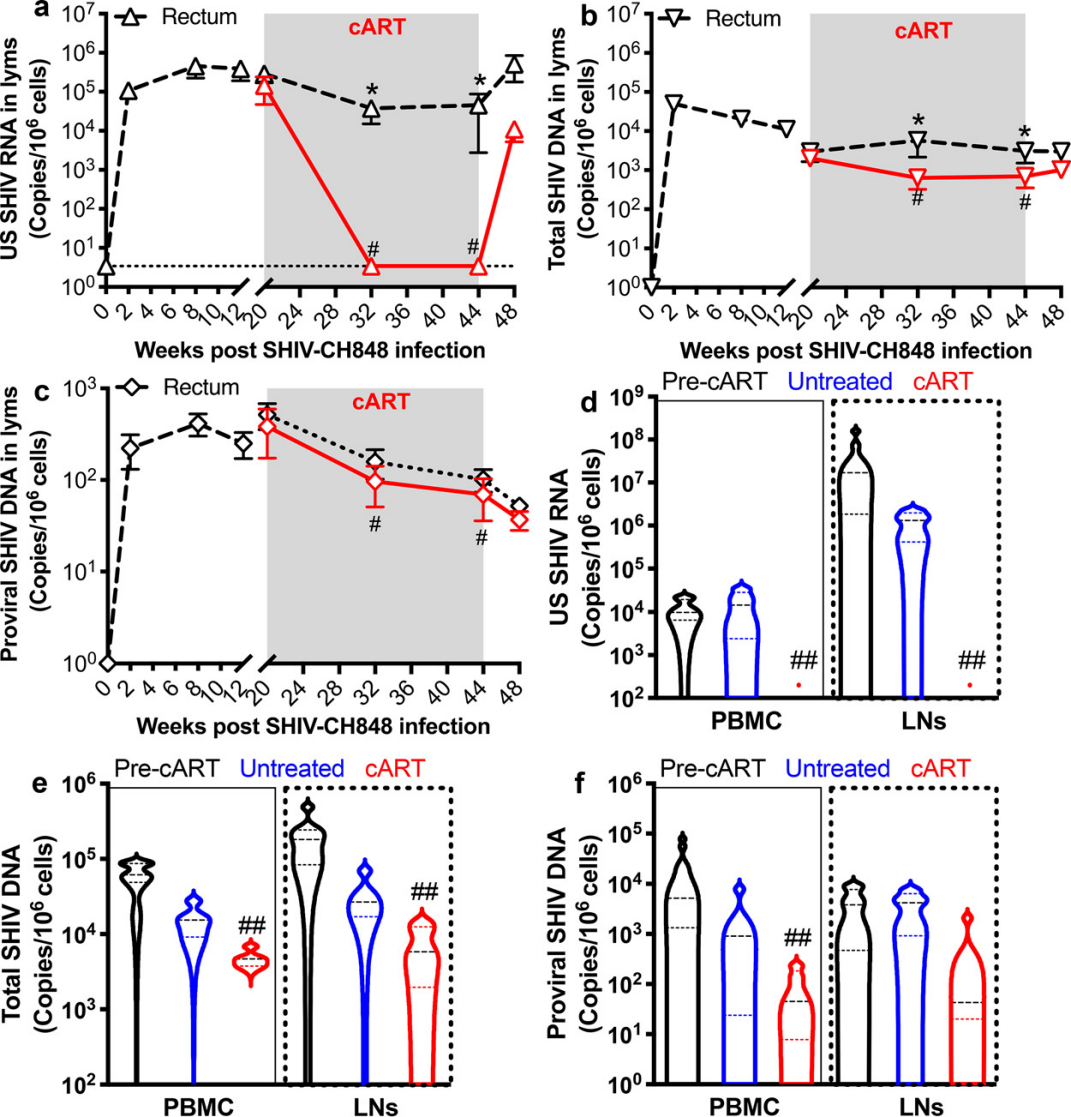
Cell-associated SHIV RNA and DNA in total PBMCs and rectal lymphocytes in SHIV.C.CH848-infected rhesus macaques on antiretroviral therapy. (a to c) Changes in levels of US SHIV RNA (a), total SHIV DNA (b), and proviral SHIV DNA (c) in rectal lymphocytes. (d to f) Levels of US SHIV RNA, total SHIV DNA, and proviral SHIV DNA in PBMCs at pretreatment (month 5 p.i.) and in cART-treated and untreated groups at 6 months. Error bars indicate SEM. *, *P* < 0.01 (paired *t* test was used to compare groups); #, *P* < 0.01 (comparison between pretreatment [month 5 p.i.] and after treatment in rectal tissue at different time points); ##, *P* < 0.05 (comparison with pretreatment or untreated controls in blood).

Cell-associated SHIV RNA/DNA was also measured from rectal lymphocytes in SHIV.C.CH848-infected macaques, with or without cART. As shown in Fig. 3a to c, the dynamics of rectal lymphocyte-associated SHIV RNA/DNA were very similar to those in blood and LN-derived CD4^+^ T cells, and cART did not significantly reduce levels of rectal cell-associated proviral DNA. Cell-associated viral nucleic acids were compared prior to treatment (5 months postinfection) and after 6 months of cART (Fig. 3d to f). Combined, these results showed that changes in SIV RNA/DNA were similar in lymphocytes from the blood, LNs, and rectum and that SHIV.C.CH848 infection results in viral persistence and stable, latent viral reservoirs in both systemic and lymphoid tissue compartments on treatment, which result in viral resurgence after treatment withdrawal.

### Plasma neutralizing antibody responses in SHIV.C.CH848-infected macaques.

We next performed TZM-bl neutralization assays to characterize neutralizing antibody responses in these macaques at 5, 15, and 24 months postinfection (Table 1). These animals developed autologous neutralizing antibody against SHIV.C.CH848 with variable potency (50% inhibitory dilution [ID_50_] titers ranged from less than 1:50 to more than 1:300) at 5 months postinfection, consistent with previous reports that autologous neutralizing antibody responses are common and arise relatively early in viremic macaques in the first few weeks to months postinfection (9, 20–22). At 2 years postinfection, regardless of cART, the plasma neutralization titers against SHIV.C.CH848 increased in 6 of 9 macaques (animal number 9 died before this time point), exhibiting neutralization ID_50_ titers of ≥1:300.

**TABLE 1:** Plasma neutralization ID_50_ titers of SHIV.C.CH848-infected rhesus macaques^*a*^

Plasma ID	ID_50_ at:									
	5 mo postinfection	15 mo postinfection	2 yrs postinfection							
	CH848 (C)	CH848 (C)	CH848 (C)	Du156.12 (C)	ZM109.4 (C)	Q23.17 (A)	BG505 (A)	Yu2 (B)	JR-FL (B)	AD17 (B)
1 (cART)	<20	<25	27	ND	ND	ND	ND	ND	ND	ND
2 (cART)	*57*	*97*	214	ND	ND	ND	ND	ND	ND	ND
3 (cART)	*96*	272	316	<25	<25	<25	<25	<25	<25	<25
4 (cART)	34	508	**1,350**	<25	28	<25	<25	<25	<25	<25
5 (cART)	*57*	100	472	<25	<25	<25	<25	<25	<25	<25
6	342	215	113	<25	<25	<25	<25	<25	<25	<25
7	149	554	392	<25	*51*	336	26	<25	26	<25
8	21	183	347	<25	<25	36	<25	<25	<25	<25
9	22	267	ND	ND	ND	ND	ND	ND	ND	ND
10	48	369	630	<25	<25	<25	<25	<25	<25	<25

a Plasma samples from SHIV.C.CH848-infected RMs at 5 months, 15 months, and 2 years postinfection were tested against a panel of autologous and heterogeneous viruses in TZM-bl cells. Clades are indicated in parentheses. Potency of 50 to 99 is indicated by italics; 100 to 999, underlining; and ≥1,000, underlining and boldface type. ND, not detected.

In contrast, none of the animals developed heterogenous neutralizing antibodies against the seven heterologous HIV-1 strains tested across clades A, B, and C at 15 months postinfection. At 2 years postinfection, there were no appreciable cross-reactive neutralizing antibodies in the 6 animals with CH848 ID_50_s of ≥1:300, except for animal number 7, in which the plasma cross-neutralized the clade A strain Q23.17 with an ID_50_ of ∼1:300 and weakly neutralized the clade C strain ZM109.4 with an ID_50_ of ∼1:50 (Table 1). In summary, 9 out of 10 SHIV.C.CH848-infected macaques mounted potent autologous neutralization antibody responses against the CH848 Env, whereas there were limited heterologous antibodies elicited in 1 infected animal.

### Viral diversity in SHIV.C.CH848-infected macaques.

**FIG 4:**
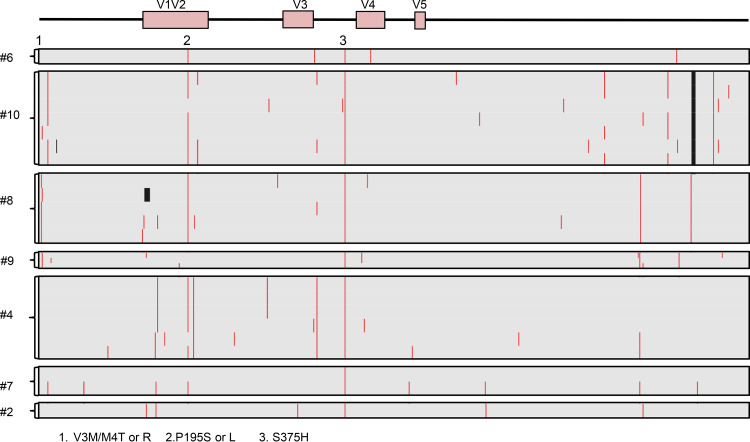
Viral Env evolution in SHIV.C.CH848-infected macaques. Shown are amino acid highlighter plots showing single-genome *env* sequences from SHIV.C.CH848-infected RM at 5 months postinfection. Nonsynonymous substitutions compared to the TF sequence are indicated by a red line, and deletions are shown as a black line. Each horizontal line shows one single-genome sequence. Two common sites of selection pressure (Env residues 3/4 and 195) were identified in infected animals.

Single-genome sequencing (SGS) of SHIV.C.CH848 gp160 *env* was used to characterize sequence diversity 5 months postinfection. A total of 31 sequences (median = 5 sequences per animal) were generated from 7 infected rhesus macaques. Sequences were aligned to the human CH848 T/F Env. As indicated in Fig. 4, 2 conserved sites of selection pressure were identified in Env: V3M or M4T/R mutations were detected in 3 of 7 macaques, and the P195S/L mutation was detected in 4 of 7 macaques. Further, while sites of selection pressure across V1V2 were identified in all 7 animals, no mutations were conserved across the entire cohort. These results suggest that at 5 months postinfection, there was minimal sequence evolution in Env in this cohort of SHIV.C.CH848-infected rhesus macaques.

## DISCUSSION

SHIVs have been widely used to explore HIV transmission, pathogenesis, latency, and cure strategies in NHP models of HIV/AIDS for over 20 years. In this study, we characterized the key features of a novel T/F SHIV.C.CH848 infection in rhesus macaques on cART. Our results indicated that SHIV.C.CH848 infection leads to the recapitulation of key immunological and virological characteristics that are hallmarks of HIV-1 infection.

HIV infection establishes a long-lived latent reservoir extremely early after infection (23). However, combined antiretroviral therapy (cART) basically fails to eliminate HIV latency characterized by integrated intact viral genomes, allowing the virus to persist for the lifetime of people living with HIV-1 (24–27). SHIV strains are very useful in NHP models for testing antiviral drugs, HIV vaccines, and functional cure strategies, especially if a small number of replication-competent viral reservoirs/latency are maintained. Although several SHIVs have been used in macaques (7, 28), their low viral persistence and often spontaneous clearance in macaques limit their potential for studying HIV latency in an NHP setting (6, 11). Recently developed CCR5-tropic SHIV.C.CH848 and SHIV.CH505 strains, encoding Env from a transmitted founder HIV-1 subtype C strain, with an increased affinity for rhesus CD4 have shown promise for viral replication kinetics more closely resembling those of HIV infection in humans (16, 17, 29). In this study, we analyzed neutralizing antibody responses and cellular reservoirs in systemic and lymphoid tissues of rhesus macaques infected with T/F SHIV.C.CH848, before and after cART treatment and interruption. These data demonstrate that this novel T/F SHIV.C.CH848 clone is promising as a candidate for testing HIV treatment and cure strategies.

T/F SHIV.C.CH848 infection in rhesus macaques during early and chronic infection closely mirrors HIV infection, as indicated by high peak viremia in primary infection, relatively stable viral set point of 10^4^ to 10^5^ viral RNA copies/ml of plasma, massive gut-associated mucosal CD4^+^ T cell depletion, stable proviral DNA in systemic and lymphoid tissues even after 6 months of cART, and viral rebound after treatment interruption. Recrudescence of SHIVC is observed within 4 months of treatment interruption, consistent with the viral rebound that occurs in most HIV^+^ patients after ART interruption, ranging from ∼5 days to 48 days (30, 31). However, viral rebound was not still observed in one animal at 6 months after ART cessation, compared with the stable viremic set point in untreated animals. Unlike SIV-infected macaques, which show rapid viral rebound after analytic treatment interruption, some SHIV.C.CH848-infected animals showed a delayed viral recrudescence, which might be attribute to limited chronic activation and latency reactivation, as indicated by lower levels of plasma inflammatory cytokines and chemokines (e.g., interleukin 8 [IL-8] and MIP-1β) at 3 weeks after treatment discontinuation, compared with those at pretreatment (data not shown). CCR5-tropic SHIV.C.CH848 efficiently infects rhesus macaques, resulting in persistent high levels of viremia, viral reservoir seeding, and depletion of CD4^+^ T cells, consistent with findings for SHIVAD8-infected macaques (8, 32, 33). However, circulating CD4^+^ T cells were rapidly restored in SHIV.C.CH848-infected macaques after ART was initiated. Given that SIVmac-infected macaques show higher viremia, and contain higher proportions of intact viral genome (∼84% for SIV versus 11.7% for HIV) on ART (34–36), SHIV.C.CH848, equivalent to HIV, likely shows less pathogenicity than SIVmac. In contrast to the depletion of CD4^+^ T cells, peripheral CD20^+^ B cells progressively increased throughout SHIVC infection in untreated animals, yet cART prevented B cell increases, suggesting that persistent SHIVC infection leads to immune activation and B cell hyperactivity if animals are untreated (37). To address the SHIV.C.CH848 latency, chronic activation, and latency reactivation after treatment interruption, more animals are needed for these studies.

In the HIV/SIV life cycle, the virus produces unspliced RNA (∼9 kb), which is responsible for *gag*/*pol* translation and packaging of the viral RNA genome, representing bona fide viral replication (38, 39). Further, integrated proviral DNA is the reliable marker of persistent viral reservoirs with clinical relevance, especially in patients when plasma viremia is undetectable (27, 40–45). Since proviral DNA is a fundamental constituent of the latent reservoir, measurement of this in cells is a simple approach to estimate the persistent cellular reservoir that may fuel viral rebound, although this method cannot distinguish defective forms. Considering that CD4^+^ T cells are primary targets and predominant cell reservoirs in HIV/SIV infection (46–48), purified CD4^+^ T cells and non-CD4^+^ cells were longitudinally compared for cell-associated viral RNA/DNA in blood and lymph nodes. Although levels of SHIV RNA/DNA were significantly different between CD4^+^ and CD4-negative cell populations, the latter including monocytes and other B and T cell lineage-negative cell subsets, the dynamics of viral RNA/DNA in two cell populations were similar, although CD4-negative cell populations showed levels of SHIV RNA/DNA that were at least 1 log lower.

Since non-CD4 cells can serve as reservoirs, including monocytes, dendritic cells, and macrophages (49–54), CD14^+^ myeloid cells were also purified to compare levels of viral RNA/proviral DNA from chronically SHIV.C.CH848-infected macaques without treatment. As suspected, viral RNA/DNA levels in purified CD4^+^ T cells were much higher than in peripheral CD4^−^ CD14^+^ myeloid cells (containing monocytes and macrophages) (viral RNA, 8.9 × 10e5 versus 6.8 × 10e4; proviral DNA, 5.8 × 10e3 versus 39.57), supporting the concept that CD4^+^ T cells are the major cellular reservoirs in HIV/SIV infection but also that myeloid cells are an additional source of viral persistence (55). CD4^+^ T cells constitute the predominant reservoir in HIV infection, yet recent advances highlight the existence of HIV reservoirs in tissue-resident myeloid cells (52, 53, 56–58). However, myeloid cells in the blood and colon likely contain HIV transcripts but few proviruses in a large fraction of HIV^+^ patients, compared with CD4^+^ T cells with readily detectable proviral DNA (59, 60). Myeloid cells, including monocytes and macrophages, are infected by HIV/SIV, while circulating monocytes might not be considered reservoirs due to their infrequent HIV infection, low levels of proviral DNA, and short life span (60). Macrophages likely represent long-lived myeloid reservoirs for viral persistence, viral rebound, and reestablishment of productive HIV infection when treatment is interrupted (61–67). Interestingly, the decay of both SHIV RNA and DNA in lymph nodes was slower than in blood under cART, consistent with the notion that lymphoid tissues may have suboptimal anti-HIV drug concentrations and thus serve as sanctuary sites, resulting in viral persistence and viral resurgence after treatment is discontinued (68–71). Importantly, cART did not significantly reduce levels of proviral DNA in blood, LNs, and rectum, suggesting that proviruses exist in multiple tissue sites, which may need to be addressed in HIV cure strategies. As our and other studies report that GC Tfh cells are expanded and impaired in chronic SIV/HIV infection (72–74), LN-derived Tfh cells also increased in untreated SHIV.C.CH848-infected animals yet decreased when cART was initiated.

People living with HIV often generate autologous neutralizing antibodies against transmitted/founder viruses within weeks to months postinfection. Similarly, high levels of autologous neutralizing antibodies were observed against SHIV.C.CH848 in 6 of 9 animals postinfection (ID_50_ titers ≥1:300). Studies indicate that the appearance and persistence of neutralizing antibodies are mostly determined by the duration of HIV/SIV infection (75, 76), yet heterogenous neutralizing antibodies were essentially undetectable after 2 years of SHIV.C.CH848 infection. This is also consistent with previous reports and suggests that the quality of GC Tfh cells and subsequent maturation of antibody responses may be compromised (77, 78). GC Tfh cells represent a subset of CD4^+^ T cells that mainly reside in the GC of follicles and involve iterative interaction with GC B cells in the GC reactions for neutralizing antibody generation (79). However, GC Tfh cell loss or functional impairment, in spite of accumulation of GC Tfh cell at the chronic stage while serving as a major source of the latent and productive viral reservoirs in persistent HIV/SIV infection, might be associated with defective Ab responses (68, 74, 80, 81). Further, virus evolution in HIV-infected patients occurs concomitantly with the emergence of selective pressures such as host CD8 T cell and antibody responses in the first weeks postinfection (82, 83), as described in the report of the source patient infected with the SHIV.C.CH848 T/F isolate, who developed first autologous and then heterologous V3-targeting antibodies (15). However, conserved sites of selection pressure in V3 were not observed in any animal 5 months postinfection, which may have been too early to generate Env mutations in response to cell or neutralizing Ab responses.

In summary, here we characterize immunological and virological events in rhesus macaques infected with the novel T/F SHIV.C.CH848, in combination with cART and in response to treatment withdrawal. These findings demonstrate that this novel T/F SHIV.C.CH848 clone has promising applications in NHP models to address questions with regard to HIV reservoirs and persistence as well as relevant HIV vaccine and cure strategies.

## MATERIALS AND METHODS

### Ethics statement.

All animals in this study were housed at the Tulane National Primate Research Center in accordance with the Association for Assessment and Accreditation of Laboratory Animal Care International standards. All studies were reviewed and approved by the Tulane University Institutional Animal Care and Use Committee under protocol number P0305R. Animal housing and studies were carried out in strict accordance with the recommendations in the *Guide for the Care and Use of Laboratory Animals* of the National Institutes of Health (NIH, AAALAC number 000594) (84) and with the recommendations of the Weatherall report *The Use of Nonhuman Primates in Research* (85). All clinical procedures were carried out under the direction of a laboratory animal veterinarian. All procedures were performed under anesthesia using ketamine, and all efforts were made to minimize stress, improve housing conditions, and provide enrichment opportunities (e.g., objects to manipulate in cage, varied food supplements, foraging and task-oriented feeding methods, and interaction with caregivers and research staff).

### Animals and virus.

A total of 10 adult Indian-origin rhesus macaques (Macaca mulatta; RMs) were intravenously inoculated with 1,000 50% tissue culture infective doses (TCID_50_) of SHIV.C.CH848 (1:10 diluted stocks containing 4.4 × 10^6^ infectious units as determined by TZM-bl cells), in which SHIV.C.CH848.375H.dCT was constructed and generated as described previously (16). After 20 weeks, 5 animals received combined antiviral drugs (tenofovir [TFV] at 20 mg/kg of body weight/day, emtricitabine [FTC] at 30 mg/kg/day, and dolutegravir [DTG] at 2.5 mg/kg/day) for 6 months. TFV and FTC were kindly provided by Gilead, Inc., and DTG was kindly provided by ViiV Healthcare. Blood, lymph node, and rectal biopsy specimens were collected at the time scheduled, processed into single-cell suspensions, analyzed by flow cytometry, and examined by quantitative cell-associated viral DNA/RNA analysis.

### Cells and plasmids.

The TZM-bl cells and the HIV-1 SG3Δenv backbone were obtained from the NIH AIDS Reagent Program (86, 87). The HIV-1 clade A, B, and C reference *rev-env* expression plasmids were obtained from the NIH AIDS Reagent Program (88–91). HIV-1 Env pseudoviruses were generated by cotransfecting 293T clone 17 (ATCC, Manassas, VA) with Env plasmids, including the parental HIV-1 clade C CH848 *rev-env* expression plasmid, along with the SG3Δenv backbone.

### Tissue collection and phenotyping.

Flow cytometry for surface and intracellular staining was performed using standard protocols (92). Cells were stained with CD3 (SP34), CD4 (OKT4; BioLegend), CD8 (SK1), CD20 (2H7), and the LIVE/DEAD fixable aqua dead cell stain kit (Invitrogen, Grand Island, NY). Isotype-matched controls were included in all experiments. All antibodies and reagents were purchased from BD Biosciences Pharmingen (San Diego, CA) unless otherwise noted. Samples were resuspended in BD stabilizing fixative (BD Biosciences) and acquired on a FACS FORTESSA (Becton, Dickinson). Data were analyzed with FlowJo software (Tree Star, Ashland, OR).

### Measurement of *gag*-specific CD8^+^ T cells in blood.

SHIV Gag-specific CD8^+^ T cells were detected as we previously described (93). In brief, PBMCs were stimulated by a pool of 15-mer Gag peptides (5 μg/ml of each peptide), medium (negative control), or phorbol-12-myristate-13-acetate (PMA; 5 ng/ml; Sigma) plus ionomycin (50 μg/ml) (positive control) for 6 h. The cultures also contained brefeldin A (Sigma) and 1 μg/ml of anti-CD49d and anti-CD28 costimulatory molecules (BD Biosciences). Cultured cells were stained with monoclonal antibodies specific for surface molecules (CD3, CD4, CD8, and LIVE/DEAD cell staining kit). After fixation and permeabilization with Cytofix/Cytoperm solution (BD Biosciences), cells were further stained with antibodies specific for gamma interferon (IFN-γ; clone 4S.B3) and TNF-α (clone MAB11) and washed with Perm/Wash buffer (BD Biosciences). Finally, labeled cells were fixed in 1.5% paraformaldehyde and acquired with a FACSA Verse cytometer (Becton Dickinson, San Jose, CA), and data were analyzed using FlowJo software (Tree Star, Ashland, OR). The background level of cytokine staining varied within different samples and different cytokine patterns but was typically <0.05% of total CD8^+^ T cells (median, 0.01%). Only samples in which the percentage of cytokine-staining cells was at least twice that of background were considered positive.

### Purification of CD4^+^ T cell subsets.

Fresh PBMCs or LN mononuclear cells (LNMCs) were incubated with anti-CD4 microbeads (Miltenyi) for 30 min, washed, and resuspended in magnetically activated cell sorting (MACS) buffer to purify CD4^+^ T cells following the manufacturer’s instruction.

### Genomic DNA and total RNA extraction.

Fresh single-cell suspensions isolated from EDTA-treated venous blood by density gradient centrifugation with lymphocyte separation medium (MP Biomedicals, Santa Ana, CA) and lymph nodes at different time points were processed to extract total genomic DNA and cellular RNA using the AllPrep DNA/RNA minikit (Qiagen) according to the manufacturer’s instructions. Viral RNA in plasma was directly isolated using the QIAamp viral RNA minikit (Qiagen). The extracted cellular DNA and RNA samples were stored at −80°C for use.

### Quantification of plasma viral load and cell-associated SHIV RNA.

The extracted RNA was reverse transcribed into cDNA using the SuperScript III first-strand synthesis system (Invitrogen) according to the manufacturer’s protocol. RT reactions were performed in a thermocycler at 25°C for 5 min and 50°C for 60 min, followed by an enzyme inactivation step at 70°C for 15 min. For quantification of targets, all primer/probe sets were synthesized by Integrated DNA Technologies (IDT; Coralville, IA) to target the SIVmac239 *gag* region (forward primer, GTC TGC GTC ATC TGG TGC ATT C; reverse primer, CAC TAG GTG TCT CTG CAC TAT CTG TTT TG; and probe, 6-carboxyfluorescein [FAM]-CTT CCT CAG TGT GTT TCA CTT TCT CTT CTG CG-BHQ-1). Plasma viral loads were measured by real-time PCR as we previously described (68). cDNA from cell-derived RNA was used to quantify unspliced RNA transcripts by digital droplet PCR (QX100 droplet digital quantitative PCR [qPCR] system; Bio-Rad) as recently described (94). Samples were run in duplicate in a 20-μl volume containing Supermix, 250 nM primers, 900 nM probe, and 2 μl of undiluted cDNA under the following cycling conditions: 10 min at 95°C, 40 cycles of 94°C for 30 s and 63°C for 60 s, and then 98°C for 10 min. Droplets were analyzed by the QuantaSoft software in the absolute quantification mode. Copies of SIV transcripts expressed as copies per 1 million cells were measured and normalized to cellular input, as determined by copies of genomic CCR5 (single-copy rhesus macaque CCR5 DNA per cell) (95–99). The limit of detection (LOD) was based on three or more replicates and calculated using GenEx 5 (MultiD Analyses AB).

### Quantification of cell-associated SHIV DNA.

To ensure that quantifications of total SHIV DNA and proviral DNA were comparable, a series of specific standards (plasmids containing SIV U5 DNA or CCR5 DNA fragment) were prepared to perform nested PCR. Since HIV preferentially integrates into regions of the chromosome close to Alu repeats, two Alu primers were used to amplify the segments of integrated proviral DNA (100). Two-step PCR amplification was run in parallel to quantify viral DNA as described previously (94). Briefly, the preamplification reactions were performed using SIV long terminal repeat primer and two outward Alu primers or using primer pairs of U5 (forward primer, AGG CTG GCA GAT TGA GCC CTG GGA GGT TC; reverse primer, CCA GGC GGC GAC TAG GAG AGA TGG GAA CAC; and probe, FAM-TTC CCT GCT AGA CTC TCA CCA GCA CTT GG-BHQ-1) on 7900HT sequence detectors (Life Technologies). The reactions were performed as follows: 25 μl of the reaction mixture, containing 1× PCR buffer, 0.2 mM deoxynucleoside triphosphates (dNTPs), 2 mM MgCl_2_, 0.8 μM each primer, and 0.5 U of *Taq* DNA polymerase (Invitrogen Life Technologies), was programmed to perform a 5-min hot start at 95°C, followed by 20 cycles of denaturation at 95°C for 30 s, annealing at 63°C for 30 s, and extension at 72°C for 3 min. Volumes of 2.5 μl of these amplicons were further amplified in triplicate with each primer/probe pair by real-time PCR using 40 cycles at 95°C for 15 s and 63°C for 1 min. The highly reproducible calibration curves were generated by plotting quantification cycle (*C_q_*) values against log-transformed concentrations of serial standard. Internal standard curves were also generated using the known copy number of target plasmids (1 to 500 copies) diluted in cellular DNA from SIV-naive RMs. The calibration curves and the internal regression curves were used for interpolating initial copies of each target in unknown samples. A nontemplate control (NTC) and extracted cellular DNA from the HUT78/SIVmac239 cell line (positive control) were included in the qPCRs. As described above, quantification of SHIV RNA/DNA was expressed as copies per 1 million cells, in which cell numbers were determined by copies of genomic CCR5 DNA per cell.

### Antibody neutralization assays.

TZM-bl neutralization assays were performed using single-round infection of TZM-bl cells with Env pseudoviruses as described previously (16, 101–103). For each of the 10 SHIV.C.CH848-infected rhesus macaques, plasma neutralization was tested at 3 time points: 5 months, 15 months, and 2 years postinfection. Briefly, TZM-bl cells were seeded at 10,000 per well. After 24 h, plasma was serially diluted 5-fold, starting at a dilution of 1:20, and incubated with 4,000 IU of virus stock as measured via titration on TZM-bl cells. The sham medium was used in place of plasma in specified control wells. The autologous infectious molecular SHIV clone, SHIV.C.CH848, and the pseudotyped HIV-1 CH848 were used to assess the neutralizing antibody titers. Pseudotyped murine leukemia virus (MLV) was used as a negative control. Antibody-virus mixtures were coincubated for 1 h and then added in triplicate to preseeded TZM-bl cells. After 48 h, cells were simultaneously lysed and mixed with luciferase substrate via the addition of Bright-Glo (Promega). Background-corrected luciferase activity for each sample was determined. Neutralization curves were fitted by the 5-parameter nonlinear regression built in Prism 8.0. The 50% inhibitory dilution (ID_50_) values were determined by the plasma reciprocal dilutions required to inhibit viral infection by 50%.

### Viral sequencing.

Single-genome full-length gp160 *env* sequences were generated as described previously (16). Briefly, up to 20,000 viral RNA copies were extracted from 400 μl of plasma virus from 5 months postinfection using the Qiagen BioRobot EZ1 workstation with EZ1 virus minikit v2.0 (Qiagen). Eluted vRNA was subsequently used as a template for cDNA synthesis and reverse transcribed using the reverse primer SHIV.Env.R1 (5′-TAC CCC TAC CAA GTC ATC A-3′) and SuperScript III reverse transcriptase (Invitrogen Life Technologies). cDNA was serially diluted in a 96-well plate (Applied Biosystems) to identify the dilution at which <30% of wells contained PCR amplicons of the correct size. The SHIV gp160 *env* genome was amplified via nested PCR with primers as follows: first-round forward primer SHIV.Env.F1 (5′-CGA ATG GCT AAA CAG AAC A-3′), second-round forward primer SHIV.Env.F2 (CTA CCA AGG GAG CTG ATT TTC), first-round reverse primer SHIV.Env.R1 (5′-TAC CCC TAC CAA GTC ATC A-3′), and second-round reverse primer SHIV.Env.R2 (5′-TAT TTT GTT TTC TGT ATGCT-3′). PCR conditions were as follows for the first round of nested PCR: 94°C, 2 min; 37 cycles of 94°C for 20 s, 55°C for 30 s, and 68°C for 3 min 30 s, and then 68°C for 10 min. For the second round of nested PCR, the PCR conditions were as follows: 94°C for 2 min; 42 cycles of 94°C for 20 s, 54°C for 30 s, and 68°C for 3 min 30 s, and then 68°C for 10 min. Amplicons were sequenced via the MiSeq platform (Illumina). Raw reads were aligned to the SHIV.C.CH848 T/F reference using Geneious R9. Sequences that contained mixed bases at a frequency of >25% per nucleotide position were excluded from further analysis. Single-genome sequences were not able to be generated for 3 of 10 rhesus macaques, likely due to a sample storage issue.

### Statistics.

Statistical analyses were performed by GraphPad Prism 7.0 software (GraphPad). Nonparametric tests were used for all statistical comparisons within animal tissues under cART. The Mann-Whitney test was used to test for differences in set point viral loads, cell-associated SHIV RNA/DNA, and specific cell subsets in animals before and after cART. Significant statistic differences (*P* < 0.05) are indicated in figures with asterisks. The data are presented as means and standard errors of the means (SEM).
